# Hemodynamic Surveillance of Ventricular Pacing Effectiveness with the Transvalvular Impedance Sensor

**DOI:** 10.1155/2014/307168

**Published:** 2014-08-04

**Authors:** Valeria Calvi, Giovanni Pizzimenti, Marco Lisi, Giuseppe Doria, Ludovico Vasquez, Francesco Lisi, Salvatore Felis, Donatella Tempio, Alfredo Virgilio, Alberto Barbetta, Franco Di Gregorio

**Affiliations:** ^1^Arrhythmology OU, Ferrarotto Hospital, University of Catania, Catania, Italy; ^2^Arrhythmology OU, Cardiology Department, Fogliani Hospital, Milazzo, Messina, Italy; ^3^Arrhythmology OU, Cardiology Department, Cannizzaro Hospital, Catania, Italy; ^4^Cardiology Department, Garibaldi-Centro Hospital, Catania, Italy; ^5^Clinical Research Unit, Medico Spa, Rubano, Padova, Italy

## Abstract

The Transvalvular Impedance (TVI) is derived between atrial and ventricular pacing electrodes. A sharp TVI increase in systole is an ejection marker, allowing the hemodynamic surveillance of ventricular stimulation effectiveness in pacemaker patients. At routine follow-up checks, the ventricular threshold test was managed by the stimulator with the supervision of a physician, who monitored the surface ECG. When the energy scan resulted in capture loss, the TVI system must detect the failure and increase the output voltage. A TVI signal suitable to this purpose was present in 85% of the tested patients. A total of 230 capture failures, induced in 115 patients in both supine and sitting upright positions, were all promptly recognized by real-time TVI analysis (100% sensitivity). The procedure was never interrupted by the physician, as the automatic energy regulation ensured full patient's safety. The pulse energy was then set at 4 times the threshold to test the alarm specificity during daily activity (sitting, standing up, and walking). The median prevalence of false alarms was 0.336%. The study shows that TVI-based ejection assessment is a valuable approach to the verification of pacing reliability and the autoregulation of ventricular stimulation energy.

## 1. Introduction

The automatic adaptation of ventricular pacing energy to the individual capture threshold has been available in the cardiac stimulation practice for the last two decades. Different systems have been proposed by the industry to prevent unnecessary high pacing output, based either on periodic threshold assessment performed by the implanted device [1, 2] or on capture check at every paced beat [3, 4]. The latter approach offers the additional advantage of continuous surveillance of pacing effectiveness, which increases the patients' safety and allows the tracking of threshold changes keeping the pulse amplitude slightly above the minimum required for cardiac stimulation. In case of capture loss, a high-energy back up pulse is delivered with a short delay from the failing stimulus [4], so that the electromechanical activity of the heart is promptly restored.

In beat-by-beat capture check as well as periodic threshold measurement, the confirmation of capture relies on the detection of pacing-induced active myocardial depolarization, that is, the action potential generated by excited myocardial fibers in the surrounding of the stimulating electrode, generally referred to as evoked potential. The evoked electrical response must be discriminated from the electrode polarization produced by the spike itself [5–8], which can be demonstrated by delivering an ineffective stimulus in the cardiac refractory period induced by a previous successful pulse [6]. Low-polarization electrodes [9, 10] and special pacing techniques [6, 8] have been developed to minimize the polarization artifact. Thanks to progressive technological improvement, the reliability of evoked potential detection has become very high and the risk of pacing failure undersensing was deemed negligible in most clinical studies [3, 4, 11, 12]. In contrast, undersensing of a positive response is still possible, especially in the event of fusion beats, and can result in undue release of the backup pacing pulse and consequent waste of energy [3, 4, 13].

In spite of the good performance of capture recognition systems based on the evoked potential detection, alternative strategies can be considered. In particular, the presence of cardiac activity could be assessed at the mechanical level, rather than from the electrical response [2]. In this way, the hemodynamic function of the heart could be monitored in any condition, instead of ventricular pacing only. A possible electromechanical dissociation, which might occur in the event of early PVCs or tachycardias, would be revealed. A false inhibition of the pacing function, due to electrical oversensing of myopotentials or electromagnetic interference from external sources, could be prevented by checking the relationship between electrical and mechanical sensing events. The confirmation of capture after ventricular pacing would thus be just a part of a general system of ejection surveillance, operating after ventricular sensing as well.

The ejection surveillance at every beat must rely on an effective haemodynamic sensor, suited to drive the pacing device in almost real time. In this aim, the measurement of cardiac impedance in transvalvular configuration (TVI) has been proposed as a tool for timely ejection recognition after either ventricular sensing or pacing [14]. TVI is derived throughout the cardiac cycle and increases when ejection occurs, decreasing back to the diastolic value as a result of ventricular filling [15–18]. Thanks to a very high signal to noise ratio, the systolic TVI rise can be used as a sensitive ejection marker. Specific algorithms have been designed to detect the TVI response and accordingly regulate the pacing function, including the pulse energy. In addition, the TVI signal is derived and displayed during a follow-up session and can be applied to drive a ventricular pacing threshold test. The present study was carried out to evaluate the performance of the TVI system in ventricular capture surveillance and detection of pacing failure during the threshold analysis, as a probe to indirectly assess TVI reliability as ejection indicator also in daily operating conditions.

## 2. Materials and Methods

The study was performed during routine follow-up checks in 4 cardiac stimulation centers in Sicily, involving 142 patients implanted with the DDDR pacemaker Sophòs 455 (Medico, Padova, Italy) for sick sinus syndrome and/or AV block. This pacemaker is endowed with the TVI sensor and the related functions of ejection surveillance after ventricular pacing and sensing and can display the telemetric TVI waveform during a threshold test controlled by TVI. The standard implantation procedure was followed, leaving the implanting physician totally free to choose the preferred pacing leads. The atrial lead was placed in right appendage and the ventricular lead in right apex. The final location was set based on threshold and intrinsic electrogram measurement, while no effort was devoted to the optimization of the TVI signal, which was not recorded at implantation.

### 2.1. TVI System Operation

The TVI system was enabled during a follow-up session scheduled at 2 months from the implantation. The signal can be derived between the atrial ring electrode and either the ring or tip ventricular electrode. Once the recording configuration is chosen, the pacemaker automatically sets the current intensity required for optimal impedance resolution, up to a maximum of 45 *μ*A. Impedance is defined by the ratio between voltage and current. The TVI detector performs this measurement with 16 ms interval, throughout the cardiac cycle. In each cycle, the voltage samples are processed by the device in order to work out the minimum TVI in the presystolic interval and the maximum TVI during the following systolic interval. All samples are displayed during waveform telemetry, while data analysis by the pacemaker starts 100 ms after the spike emission (a blanking period set to avoid artifact sensing). The difference between maximum and minimum TVI assessed after the blanking period provides the peak-to-peak TVI excursion (TVI_ pk-pk _). To allow the system application in ejection surveillance, the TVI signal must reach or exceed a minimum TVI_ pk-pk_ and satisfy the expected time-course, in which the minimum presystolic TVI precedes the systolic maximum. Therefore, as a preliminary step, the basic properties of the TVI signal in the patient group were assessed and compared in the presence of AV sequential pacing (VDD if the intrinsic atrial rate was not lower than 50 bpm and DDD at 60 bpm otherwise, setting the AV delay to be short enough to induce a fully evoked QRS on the surface ECG) and VVI stimulation at overdrive rate (the pacing mode usually applied in threshold measurement). The significance of differences in TVI_ pk-pk_ related to the pacing modality was evaluated by the paired Student's *t* test. The spread of the data is always expressed as standard deviation.

### 2.2. TVI in Ventricular Capture Assessment

In case of ventricular pacing, capture is confirmed if TVI_ pk-pk_ in the current cycle is equal or higher than 50% of the reference value, which corresponds in turn to the latest updating of the average TVI_ pk-pk_ derived from 8 positive TVI signals. If this condition is not satisfied during standard operation, the pulse energy is increased and a threshold test is then carried out automatically by the pacemaker. If the condition is not satisfied during a threshold test started by the programmer within a check-up session, the procedure is broken and the permanent pulse parameters are resumed, storing in memory as threshold data the lowest amplitude and width which induced a positive TVI response.

To test TVI reliability in capture surveillance, the ventricular threshold analysis was carried out in VVI mode at overdrive pacing rate in both supine and sitting upright position. The procedure was simultaneously monitored by both the pacemaker through the TVI signal and the physician through the surface ECG. On the occurrence of TVI-indicated pacing failure, the pacemaker automatically restored the programmed pulse energy and quit the test mode. Only in the event of undetected capture loss, the threshold test must be manually aborted by the physician. When the procedure was stopped for any reason, the pacemaker diagnosis of a positive or negative response to stimulation in the last paced beat was cross-checked with the physician's evaluation, assumed as the golden standard. The test was considered successful in each patient only if TVI-based autoregulation of pulse energy scan down to the threshold was consistent with the physician's interpretation of the ECG in both supine and sitting position. The TVI sensitivity to capture failure was expressed by the proportion of patients where a true capture loss was properly recognized by TVI in both conditions, with respect to the number of patients where the threshold was reached. The positive predictive value was given by the proportion of patients where a true capture loss was properly recognized by TVI in both conditions, with respect to the total number of tested patients. Two-tailed 95% confidence limits of these indices were derived from the binomial distribution.

In addition, at the end of the check session the pacemaker was set in DDD with AV delay shorter than the intrinsic PR interval by at least 50 ms, and the ventricular pulse energy was transiently programmed at four times the threshold, in order to make virtually nil the likelihood of capture failure. The function of TVI-based ejection surveillance was set in passive mode (i.e., the ejection presence was checked at every beat without affecting the programmed pulse energy) and the patients were asked to alternate sitting and standing up positions with periods of walking at their usual speed, for a 10 to 15 min observation interval. During this period, the pacemaker stored in memory the number of cycles where a capture failure was indicated by the TVI sensor, together with the total number of ventricular pacing events and the trend of the alarm frequency as a function of the time. The data were retrieved by telemetry at the end of the test. The prevalence of TVI-indicated capture failures (assumed to represent false alarms) was considered as a negative indicator of the system specificity. The frequency distribution of the individual alarm prevalence in the patient group was summarized by its quartiles.

## 3. Results

### 3.1. TVI Basic Properties

**Figure 1:**
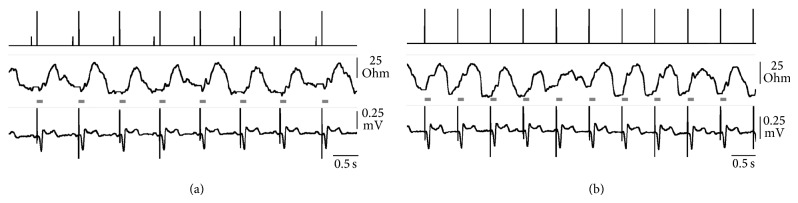
Real-time telemetry from the implanted pacemaker. Event markers (upper tracing; short bar = atrial sensing; long bar = ventricular pacing) and transvalvular impedance (TVI; middle tracing; recording in ventricular tip configuration) are matched with the surface ECG (lead II; lower tracing). The horizontal grey bars represent the TVI blanking period following the ventricular spike emission. Atrium-driven (a) and VVI stimulation (b) are compared in the same patient.

TVI was derived with the tip ventricular electrode in 52% of the patients and with the ring in the remaining 48%. When the atrial ring-ventricular tip configuration was chosen, TVI_ pk-pk_ was higher with AV sequential than VVI pacing (45.0 ± 22.3 and 39.9 ± 22.9 Ohm, resp.; *P* < 0.005). The individual ratio of TVI_ pk-pk_ in VVI and VDD averaged 0.87 ± 0.22 in this subgroup of patients. When TVI was derived in atrial ring-ventricular ring configuration, TVI_ pk-pk_ was remarkably lower and no significant difference related to the pacing mode was noticed (17.5 ± 8.4 and 16.4 ± 7.0 Ohm in AV sequential and VVI pacing, resp.). In most cases featuring a lower TVI_ pk-pk_ with VVI pacing, the difference resulted from a higher beat-to-beat variability compared with sequential activation (the coefficient of variation was 0.18 ± 0.11 and 0.13 ± 0.08, resp.; *P* < 0.05), associated with the anticipation of TVI rise. Due to the earlier signal onset, the actual minimum TVI could occur in the blanking period following the ventricular spike and thus could be skipped by the measurement of TVI excursion performed by the pacemaker. A comparison of TVI signals recorded in the same patient in VDD and VVI pacing is shown in Figure 1. In this representative case, TVI was derived by the ventricular tip electrode. In VDD, the waveform featured a gradual increase taking place mainly after the end of the blanking period, and the measured TVI_ pk-pk_ averaged 45.8 ± 3.3 Ohm with a variation coefficient equal to 0.07. In VVI, in contrast, the TVI rise started within the blanking, so that the measured TVI_ pk-pk_ was reduced to 26.8 ± 9.7 Ohm, with a variation coefficient of 0.36.

### 3.2. Ventricular Threshold Analysis

Since the ventricular threshold test was performed in VVI pacing and the quality of the TVI signal was worsened in such conditions, the system enabled the procedure under TVI control in 120 out of 142 patients (85% of the cases). The energy scan was broken by TVI for a real capture loss confirmed by the surface ECG in both supine and sitting position in 115 patients, while in 5 cases the procedure was incorrectly stopped by TVI for a false alarm of capture failure occurring in either supine or sitting position. In no case the procedure was manually closed by the physician in the event of capture failure undetected by TVI, as all 230 episodes of missing capture (115 in each postural condition) were promptly recognized by the system. TVI sensitivity to capture loss thus resulted 100% in the tested group, with 95% confidence limits ranging from 96.8 to 100% in the population. The positive predictive value was 95.8% in the sample, with 95% confidence limits from 92.3 to 99.4%.

**Figure 2:**
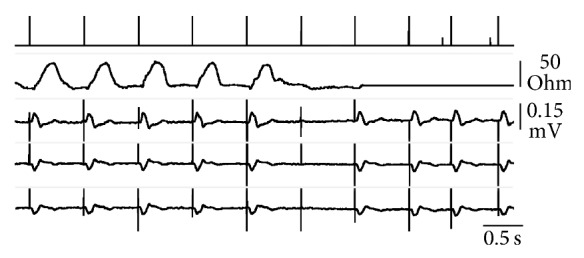
Real-time telemetry of event markers (1st tracing) and transvalvalvular impedance (2nd tracing) with simultaneous surface ECG recording (I, III, and aVR from the 3rd to bottom tracings, all with the same voltage scale) during ventricular threshold analysis in VVI. On the markers tracing, long bars represent ventricular pacing and short bars atrial sensing. The 6th spike energy was below the threshold. The capture failure was recognized by the TVI system and the pacemaker promptly increased the pulse amplitude, restoring effective ventricular stimulation.

An example of the system operation is illustrated in Figure 2, which shows a ventricular threshold analysis with overdrive VVI stimulation. When ventricular pacing was effective, a TVI signal featuring the expected increase in the QT interval was generated at every beat. In contrast, when the stimulus did not reach the threshold (6th pulse in Figure 2) the electromechanical response was absent, no ejection occurred, and therefore TVI remained at the diastolic level. The absence of a TVI increase after the ventricular spike was detected by the pacemaker, which restored the programmed pulse energy and closed the procedure, switching back to VDD from the temporary VVI pacing mode. During the reprogramming process, TVI telemetry was suspended.

**Figure 3:**
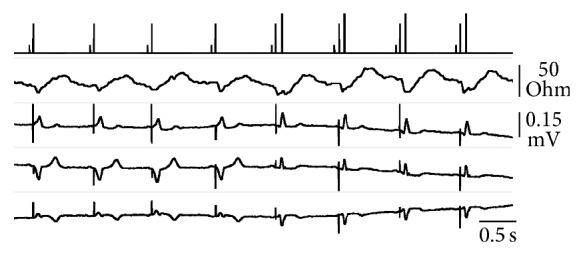
Real-time telemetry of event markers (1st tracing) and transvalvalvular impedance (2nd tracing) with simultaneous surface ECG recording (I, II, and aVR from the 3rd to bottom tracings, all with the same voltage scale) during ventricular threshold analysis in VDD. On the markers tracing, short bars represent atrial sensing, intermediate bars ventricular pacing, and the longest bars ventricular sensing in the pacemaker refractory period. From the 5th pulse onward the stimulation was below threshold and a narrow QRS replaced the pacing-evoked wide complex. Nevertheless, a properly timed TVI fluctuation was present, confirming the ejection occurrence, and the energy scan continued down to the minimum pulse amplitude available.

Although TVI proved very sensitive to the absence of mechanical activity following ventricular pacing, the system could not discriminate between different activation patterns. Therefore, the only feature suitable to distinguish intrinsic and evoked ventricular contraction was the time-relationship with the pacing spike. In few cases where the TVI signal was so deteriorated in VVI to be unacceptable to the control system, a threshold test was attempted in the presence of sequential pacing. Unless the intrinsic AV conduction was absent or very slow, a TVI fluctuation due to natural conduction was detected anyway within the expected systolic interval triggered by the pacing pulse, even with a short AV delay. As a result, properly timed ejection was confirmed by TVI and no capture-loss alarm was raised when the QRS shifted from an evoked wide complex to a narrow signal, indicating the occurrence of intrinsic conduction instead of effective stimulation. An example is provided in Figure 3.

### 3.3. Prevalence of Capture Failure Alarms

The prevalence of false alarms of capture loss during daily life activity did not exceed 0.25% of paced ventricular beats in 47% of the patients. The frequency distribution of the individual prevalence featured a 1st quartile equal to 0.047%, a median of 0.336%, and a 3rd quartile corresponding to 1.110%. This means that, on the average, 1 false alarm was produced in more than 2128 paced beats in 25% of the patients, in more than 298 beats in 50% of the patients, and in more than 90 beats in 75% of the patients.

To calculate the energy cost of this error rate, it must be pointed out that the pacing pulse parameters in Sophòs pacemakers are specified by the minimum and maximum amplitude and width, which are all programmable. As long as ventricular capture is confirmed by TVI, the minimum pulse amplitude and width are applied. Following an alarm of capture failure, the maximum amplitude and width are enabled for 8 beats; then width and amplitude are decreased in sequence down to the programmed minimum values, with a reduction speed of 1 programming step per cardiac cycle. Therefore, the increase in pacing energy induced by a single alarm depends on the programmed pacing configuration. Assuming that the maximum amplitude is twice the minimum with 12 programming steps in between and with constant pulse width, the 1st quartile alarm prevalence would imply a 1.8% increase in the current drain due to ventricular pacing. The median and 3rd quartile prevalence would result, respectively, in 12.6 and 41.6% rise in the consumption required for ventricular stimulation with respect to the minimum predicted in case of no alarms. To restrict the extraconsumption below 25% in this simulation, the alarm rate must not exceed an upper limit of 1 in 150 paced cycles, corresponding to a prevalence of 0.67%.

## 4. Discussion

Checking the regular occurrence of ventricular ejection at every beat might substantially increase the patient's safety and improve the quality of the pacing therapy. Our experience has shown that the absence of ventricular contraction after ineffective stimulation was promptly detected by the TVI system and that all the episodes of asystole were recognized and properly managed (Figure 2). The high TVI sensitivity to the lack of ventricular mechanical activity supports the use of this tool in the hemodynamic validation of the electrical sensing, in addition to capture surveillance [14]. Thanks to the total absence of false-positives, TVI-based cross-check is expected to prove to be a valuable solution in the prevention of inappropriate pacing inhibition, which might result from ventricular oversensing.

In conditions of ventricular pacing as well, TVI looks best suited to a function of ejection confirmation aimed at increasing the reliability of the pacing system, rather than to the continuous measurement of the pacing threshold required for the fine regulation of pulse energy [4, 12]. Indeed, the TVI fluctuation follows the time-course of ventricular contraction and relaxation and is therefore a slow signal, which must be sampled for some hundreds of milliseconds to state whether the ejection is taking place or not [15, 17, 18]. In case of a negative result, the diagnosis is available too late to deliver a high energy back-up pulse within the current cardiac cycle. The energy increase is therefore delayed to the next cycle at the price of a lost beat, which is absolutely acceptable provided it does not happen very often. On the other hand, modern electrode technology allows reliable cardiac pacing with low energy even if the threshold is not tracked at every beat. The essential issue is to balance the risk of low energy pulse setting with a sensitive system of capture failure detection, which can increase the pacemaker output in case of need. TVI has shown the properties required to successfully play this role.

As the TVI system has been designed to detect the ejection, no matter if it is produced by ventricular stimulation or intrinsic activity, it could be difficult to assess the pacing threshold in the presence of intrinsic AV conduction (Figure 3). However, information on the real threshold is not essential for the autoregulation of the pacing device, as long as properly timed ejection spontaneously occurs. Increasing evidence has been produced that ventricular pacing could be deleterious and should be avoided, if possible [19–22]. Therefore, the most advisable pacemaker regulation in a phase of intrinsic ventricular activity is just to stand-by, being ready to stimulate the ventricle even with high energy only in the event of conduction block. A threshold test could then be performed when ventricular pacing is required to treat a bradyarrhythmia, in order to reduce the energy expense by pacing output adaptation. Furthermore, the similar TVI response recorded after pacing or intrinsic conduction avoids the problem of undue energy increase in the presence of fusion beats, which commonly affects the performance of capture recognition systems based on the detection of electrical evoked potentials [2–4, 11, 12]. The issue has been managed by the introduction of dedicated algorithms which modify the AV delay after a back-up pulse (and related energy increase) to unmask fusion and pseudofusion [13]. The regulation of the AV delay, however, is another crucial point in the ideal setting of a pacing device [23] and should only be aimed at the optimization of ventricular filling and contraction mechanics, reducing as much as possible the incidence of changes imposed for other reasons.

The high signal-to-noise ratio generally exhibited by TVI allowed a strict definition of the positive response, which must feature a sufficiently large TVI rise taking place within a specified time interval. This strategy intentionally optimized the sensitivity to capture loss to the prejudice of specificity and general system applicability. In addition, the present experience shows that the quality of the TVI signal worsened in VVI with respect to AV sequential pacing, even if the QRS complex was very similar, indicating a fully evoked ventricular activation in both cases (Figure 1). However, VVI stimulation entails the loss of the atrial contribution to ventricular filling, which can affect cardiac hemodynamics. With this pacing mode, the TVI waveform recorded with the tip ventricular electrode often showed an early and steep rise, which made difficult the measurement of the minimum presystolic value. Since TVI is modulated by cross-section changes occurring in the ventricular region where the recording electrode is placed [17, 18], which was the right apex in the present experience, we could speculate that the movement of the stimulated ventricular myocardium is delayed by the active blood inflow, especially if the AV delay is short, or by passive stretching of the ventricular wall which might be caused by the atrial contraction. For all these reasons, the use of TVI for capture verification during VVI pacing was possible in 85% of the cases. In a previous study, the discrimination of the electrical evoked response from the passive electrode polarization was effective in 71% and 94% of the cases, respectively, by applying conventional or modified pacing pulses [6]. Other Authors reported that an evoked potential amplitude sufficient to enable the capture recognition algorithm was found in 93% of the assessed patients [5]. The present TVI applicability in capture surveillance could be increased by shortening the blanking period preceding the start of TVI processing and, most importantly, by recording the TVI signal during the implantation procedure, aiming at a lead position where the waveform is suitable for ejection detection and is mildly affected by the pacing mode. Few minutes care is supposed to be enough to substantially improve the outcome. A special pacing system analyzer (PSA), specifically designed to this purpose, has been approved for the clinical use after the end of this study.

A less demanding identification of a positive TVI response to ventricular stimulation is also expected to reduce the prevalence of capture failure alarms in daily life, with consequent decrease of the total energy used to pace the ventricle. Although the alarm rate was lower with the TVI system than in the early experience with electrical evoked potentials, where a back-up pulse was delivered in 1.1% of paced cycles mostly due to undersensing of fusion beats [3], the current TVI performance can be further improved. As the apparent sensitivity in the assessed sample was 100%, there seems to be a good margin to shift the balance of sensitivity and specificity towards an appropriate and convenient compromise.

## 5. Conclusions

At present, TVI is the only hemodynamic sensor proposed as a tool for ejection surveillance at every beat. While capture recognition based on evoked potential detection still requires the use of low polarization electrodes for full reliability [24–26], TVI can be recorded with any pacing lead. The TVI system demonstrated excellent sensitivity to capture failure during threshold analysis, coupled with an acceptable specificity. Future effort to further improve the specificity must include a more tolerant definition of a positive TVI response to ventricular stimulation and some care in lead positioning on implantation, aimed at optimizing the individual waveform, which can now be recorded by means of a dedicated PSA.

TVI-based ejection surveillance can be applied in the presence of ventricular pacing and sensing as well, and might substantially increase the safety of bradyarrhythmic patients treated with cardiac stimulation.

## 6. Limitations

The reported experience was limited to the acute evaluation of TVI performance in the detection of a capture failure and the autoregulation of the pacing energy. Chronic clinical studies are required to establish the reliability of the system and its energy cost in long-term follow-up.
